# Biallelic *BAIAP3* Variants Are Associated with Isolated Retinitis Pigmentosa

**DOI:** 10.3390/ijms26178244

**Published:** 2025-08-25

**Authors:** Viviana Cordeddu, Elisabetta Flex, Luca Mignini, Alessandro Bruselles, Serena Cecchetti, Elena Messina, Maria Beatrice Arasi, Mattia Carvetta, Emilio Straface, Alessandro Leone, Daniele Guadagnolo, Maria Cecilia D’Asdia, Marcella Nebbioso, Emanuele Bellacchio, Carmen Dell’Aquila, Lucia Ziccardi, Antonio Pizzuti, Alessandro De Luca, Marco Tartaglia

**Affiliations:** 1 Department of Oncology and Molecular Medicine, Istituto Superiore di Sanità, Viale Regina Elena 299, 00161 Rome, Italy; elisabetta.flex@iss.it (E.F.); alessandro.bruselles@iss.it (A.B.); mariabeatrice.arasi@opbg.net (M.B.A.); strafaceemilio8@gmail.com (E.S.); alessandro.leone@iss.it (A.L.); 2 Molecular Genetics and Functional Genomics, Ospedale Pediatrico Bambino Gesù, IRCCS, Viale di San Paolo 15, 00146 Rome, Italy; luca.mignini@opbg.net (L.M.); mattia.carvetta@opbg.net (M.C.); emanuele.bellacchio@opbg.net (E.B.); 3 Confocal Microscopy Unit—Core Facilities, Istituto Superiore di Sanità, 00161 Rome, Italy; serena.cecchetti@iss.it; 4 Department of Biochemical Sciences “Alessandro Rossi Fanelli”, Sapienza University of Rome, 00185 Rome, Italy; 5 Department of Humanities, Law and Economics, Telematic University Leonardo da Vinci, UNIDAV, Torrevecchia Teatina, 66100 Chieti, Italy; 6 Department of Experimental Medicine, Sapienza University of Rome, Piazzale Aldo Moro 5, 00185 Rome, Italy; daniele.guadagnolo@uniroma1.it (D.G.); antonio.pizzuti@uniroma1.it (A.P.); 7 Medical Genetics Laboratory, Fondazione IRCCS Casa Sollievo della Sofferenza, 71013 San Giovanni Rotondo, Italy; mc.dasdia@operapadrepio.it (M.C.D.); a.deluca@operapadrepio.it (A.D.L.); 8 Department of Sense Organs, Sapienza University of Rome, Piazzale Aldo Moro 5, 00185 Rome, Italy; marcella.nebbioso@uniroma1.it; 9 IRCCS-Fondazione Bietti, Via Livenza 1, 00198 Rome, Italy; carmen.dellaquila@fondazionebietti.it (C.D.); lucia.ziccardi@fondazionebietti.it (L.Z.); 10 Department of Medicine and Health Sciences “V. Tiberio”, University of Molise, Via F. De Sanctis 1, 86100 Campobasso, Italy

**Keywords:** retinitis pigmentosa, retinal dystrophy, whole genome sequencing, *BAIAP3*, ciliogenesis

## Abstract

A class of retinal dystrophies known as retinitis pigmentosa (RP) is caused by the loss of photoreceptor cells. RP can be genetically transmitted as an autosomal dominant, autosomal recessive, or *X*-linked trait. About one-third of genes implicated in retinal degeneration encode for proteins whose functional dysregulation affects the “connecting cilium” in photoreceptors, altering its structure and function. Here we report on a 33-year-old woman who was referred for clinical genetic testing following a previous diagnosis of degenerative retinopathy, which was not informative. She was enrolled in a research program dedicated to undiagnosed retinal disorders, where a whole genome sequencing approach was employed to understand the underlying genetic basis. The genomic analysis documented the occurrence of compound heterozygosity for two functionally relevant missense variants in *BAIAP3*, which encodes a protein with a well-documented role in SNARE-mediated trafficking and ciliogenesis. Confocal microscopy analysis showed elongated cilia in patient-derived and *BAIAP3*-depleted fibroblasts compared to control cells. Real-time PCR analyses showed a consistent significant reduction of *GLI1* mRNA levels in patient-derived and *BAIAP3*-depleted cells, both in basal conditions and after treatment with Smoothened agonist, SAG, indicating Sonic hedgehog signaling dysregulation. Collectively, these data suggest that biallelic loss-of-function variants of *BAIAP3* may cause photoreceptor degeneration and underlie isolated RP.

## 1. Introduction

Retinitis pigmentosa (RP, MIM #26800) refers to a group of clinically and molecularly heterogeneous inherited retinal dystrophies (IRDs) affecting approximately 1 in 3000 to 5000 individuals, and characterized by degeneration of photoreceptor cells [1]. Genetically, RP can be transmitted as an autosomal dominant, autosomal recessive, or X-linked trait [2]. RP has also rarely been described as a digenic disorder [3]. Most cases are nonsyndromic, meaning they are not associated with extraocular clinical features (i.e., nonsyndromic RP) [4]. Clinically, RP presents with progressive night blindness and loss of peripheral vision [4]. Over time, central vision and visual acuity can be involved [4]. The clinical course reflects the primary loss of rod photoreceptors (predominant in the peripheral retina, with a key role in low-light vision) [4]. Rod dysfunction can be assessed with electrophysiology assays [4]. Loss of photoreceptors eventually leads to the typical ocular fundus appearance with pigmentary deposits, but retinal vascular attenuation in the affected areas may appear before pigmentary changes [4].

The majority of the genes causally associated with IRDs encode proteins that are essential to the visual cycle and photoreceptor function [2]. This genetic heterogeneity, along with the variable expressivity of pathogenic variants, results in the considerable clinical variability characterizing RP in terms of age of symptom onset, progression rate, and pattern of retinal degeneration. Of note, dominant and recessive forms of rod-cone dystrophy can occasionally be caused by pathogenic variants in the same gene, with the dominant form typically presenting a milder phenotype [5]. Advances in molecular genetics have enabled a deeper understanding of the pathogenetic mechanisms underlying retinal pathology, providing new insights into its molecular bases.

A significant proportion of RP genes encode proteins that are essential for the structure and function of photoreceptors, particularly at the connecting cilium that links the inner and outer segments. The cilium not only provides structural support but also regulates the trafficking of phototransduction proteins and renewal of outer segment discs. Defects in ciliary proteins disrupt these processes, explaining why cilia-related genes account for nearly one-third of RP cases [6,7]. These genes can be broadly categorized according to their roles within the cilium. Structural proteins of the axoneme and basal body, such as RP1, RP1L1, FAM161A, and LCA5, maintain the integrity of the microtubular scaffold and stabilize the outer segment, while additional proteins including KIZ, C2ORF71, SPATA7, and TOPORS contribute to ciliary organization [8,9,10]. A second functional group comprises transition zone and centrosomal adapter proteins, including CEP290, RPGR, RPGRIP1, and RPGRIP1L, which regulate selective trafficking at the connecting cilium and anchor key structural elements [11,12,13]. Finally, intraflagellar transport components, such as the proteins of the IFT complex and BBSome, sustain the continuous bidirectional flow of proteins and lipids between the inner and outer segments, a process indispensable for photoreceptor survival [14,15]. As a consequence, mutations in these structural, regulatory, or trafficking proteins functionally converge on the common phenotype of progressive photoreceptor degeneration, underscoring cilia dysfunction as a unifying mechanism in RP and providing a framework for therapeutic approaches aimed at restoring ciliary stability or transport.

In RP, rod photoreceptors degenerate earlier than cones, despite many causative mutations affect shared cellular structures. This selective vulnerability of rods is primarily due to their high metabolic demand and dependence on cilia-associated proteins critical for outer segment renewal and trafficking [16]. Rods constitute approximately 95% of photoreceptors in the human retina, and their loss leads to structural disorganization of the outer nuclear layer (ONL), which impairs the physical and functional interactions between cones and the retinal pigment epithelium [17]. The delivery and uptake of key metabolites, including glucose, is also reduced [18]. The subsequent death of cones is driven by a combination of metabolic, oxidative, and inflammatory stressors [19,20]. Recent findings also implicate innate immune activation, particularly microglial reactivity, in exacerbating cone stress [21]. Additionally, unlike rods, which typically die by apoptosis, cones often undergo necroptosis—a programmed form of necrosis—suggesting the involvement of RIPK-dependent pathways [22].

*BAIAP3* (MIM 604009) encodes a protein, brain-specific angiogenesis inhibitor-1 associated protein 3, that interacts with the cytoplasmic domain of BAI1 (brain-specific angiogenesis inhibitor-1; MIM *602682, also known as adhesion G protein-coupled receptor B1), a protein negatively controlling angiogenesis [23]. BAIAP3 contains two C2 domains, which are commonly found in proteins involved in signal transduction or membrane trafficking, and a complex associated with tethering containing helical rods (CATCHR) domain, which regulates retrograde transport from endosomes to the trans-Golgi network (TGN) [24,25,26,27]. BAIAP3 interacts with SNARE proteins (e.g., STX6, STX16, VAMP3, and VAMP4), whose function is essential for endosome-mediated retrograde trafficking, in a manner similar to that described for COG and GARP protein complexes [26]. Evidence also supports a role of BAIAP3 in synaptic function. Single-cell RNA sequencing profiling showed enhanced expression of this gene in ciliated cells, astrocytes, excitatory and inhibitory neurons, and enteroendocrine cells (https://www.proteinatlas.org/ (accessed on 4 March 2025)). While pathogenic mutations in *BAIAP3* have not been identified yet, the involvement of the SNARE protein complex in human disease is well established [27].

Here, we report a sporadic case of RP that remained molecularly unexplained despite extensive genetic testing. Trio-based whole genome sequencing (WGS) identified biallelic missense variants in *BAIAP3* as the putative molecular cause. In patient-derived and *BAIAP3*-depleted fibroblasts, we show an altered biogenesis of primary cilia, which were characterized by an elongated ciliary axoneme, and defective cilium-associated Sonic hedgehog (SHH) signaling, suggesting impaired ciliary trafficking activity. Our findings provide evidence that loss of BAIAP3 underlies photoreceptor degeneration and isolated RP.

## 2. Results

### 2.1. Clinical Assessment

The patient, a 33-year-old female at the time of evaluation, was referred for clinical genetic assessment following a prior ophthalmological diagnosis of RP. She reported no direct parental consanguinity; however, both parents originated from a small, geographically isolated community of approximately 600 inhabitants. There was no family history of RP, chromosomal disorders or other clinical conditions with Mendelian inheritance. Her pedigree included a healthy older brother, aged two years her senior. The patient was born full-term following an uneventful pregnancy. Both her physical and cognitive development were normal, and her medical history, comprehensive of auditory screening, was unremarkable.

Symptoms first appeared at 18 years, presenting as night blindness and an initial, slowly progressive loss of peripheral vision. At that time, she received a clinical diagnosis of RP after ophthalmological evaluation. The condition showed slow progression over time, without significant impairment of central vision or visual acuity. At her first clinical genetics assessment, conducted at age of 33 years, physical examination revealed no abnormalities, or dysmorphic features. Previous medical records, including abdominal ultrasound imaging and echocardiography, were unremarkable. A comprehensive ophthalmological assessment was requested, including best corrected visual acuity (BCVA) measurement using the Early Treatment Diabetic Retinopathy Study (ETDRS) charts (Lighthouse, Low Vision Products, Long Island City, NY, USA) at a distance of 4 m, expressed as the logarithm of the minimum angle of resolution (logMAR). Visual field (VF) testing was performed using the Humphrey VF 30-2 threshold test (Humphrey field analyzer, HFA, Carl Zeiss Meditec, Jena, Germany). Retinal imaging encompassed spectral domain (SD) optical coherence tomography (OCT), fundus autofluorescence (FAF) by the SPECTRALIS OCT imaging platform (Heidelberg Engineering, Heidelberg, Germany), and OCT angiography (OCTA) using the Optovue Solix system (Visionix Italia S.R.L., Garbagnate Milanese, Italy). The patient also underwent scotopic, mesopic and photopic electroretinograms (ERG) using a computerized Optoelectronic Stimulator Vision Monitor (MonPack 120 Metrovision. Pérenchies, France), following the 2022 International Society for Clinical Electrophysiology of Vision (ISCEV) updated standards. Recordings were obtained using ERG-Jet corneal electrodes after pupil dilatation at 8 mm [28].

**Figure 1:**
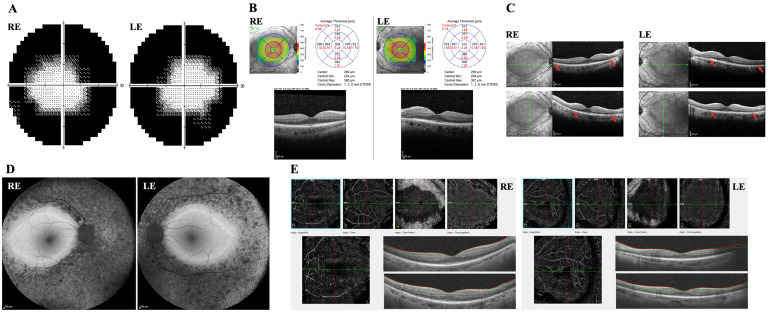
Ophthalmological instrumental assessment of the patient. (**A**) Humphrey Visual Field (VF) 30-2 Threshold Test, performed by Humphrey Field Analyzer, showed bilateral loss of peripheral sensitivity with central sparing up to 10 degrees in the temporal side and 15 degrees in the nasal side in right eye (RE) and left eye (LE) (mean deviation RE = −24.33 dB and LE = −24.02 dB). (**B**) Spectral-domain optical coherence tomography (SD-OCT) map showed macular thickness within normal limits in RE and LE. (**C**) Vertical and horizontal SD-OCT scans (30°) displayed bilateral peripheral loss of the inner/outer photoreceptor segment junction and of the photoreceptor layers, marked with red arrows, with foveal sparing. Green lines represent the scans passing through the fovea horizontally and vertically. (**D**) Retinal fundus (55°) blue-light autofluorescence (FAF) imaging showed peripheral hypo-autofluorescence with dense mottling and black spots, suggesting retinal pigment epithelium degeneration and intraretinal pigmentation. The central area displays relative sparing with hyper-autofluorescence ring delimitating the still partially functioning retina within the vascular arcades. The findings were similar and symmetrical in RE and LE. (**E**) OCT angiography (OCTA) 8 mm scans of the macula and perimacular retina showed a 360° jagged edge separating peripheral more hypoperfused choroidal areas as compared to central regions.

The ophthalmological findings were consistent with a clinical diagnosis of RP. BCVA was of 0.0 logMAR in both eyes (OU), with dioptric corrections of +0.25 sphere and +0.50 cylinder at axis 100° in the right eye (RE) and +0.50 sphere and +0.50 cylinder at axis 90° in the left eye (LE). HFA VF 30-2 threshold testing evidenced markedly reduced peripheral sensitivity (mean deviation of −24.33 dB in RE and −24.02 dB in LE), with central visual field sparing up to 10 degrees temporally and 15 degrees nasally in both eyes (Figure 1A). Bilateral dilated fundus examination showed arteriolar attenuation, retinal pigmentary changes with hyperpigmentation in the form of diffuse bone-spicule deposits and pigment clumping in the mid-peripheral retina, alongside waxy disc pallor.

Retinal imaging details are presented in Figure 1B–E. SD-OCT demonstrated normal foveal thickness in OU (Figure 1B), which was indicative of preserved outer retinal layer morphology in the foveal and parafoveal regions. Bilateral disruptions of the inner segment/outer segment (IS/OS) junction were observed, extending from the perimacular area to the retinal periphery, consistent with widespread photoreceptor loss (Figure 1C). The preservation of the foveal structure correlated with maintained BCVA and central VF sparing. FAF imaging showed diffuse peripheral hypo-autofluorescence with dense pigmentary mottling, suggesting retinal pigment epithelium degeneration and intraretinal pigmentary deposits. The central macular region, within the vascular arcades, was structurally spared, marked by a sharply delineated hyper-autofluorescent ring demarcating the boundary of retinal abnormalities. These structural findings were bilateral and symmetrical (Figure 1D).

Macular and perimacular retina OCTA imaging showed attenuation of choroidal vessels, and highlighted a 360° jagged edge demarcating the more severely affected peripheral areas from the relatively preserved central regions (Figure 1E). The SD-OCT scan assessing the retinal nerve fiber layer showed overall normal thickness, with a reduction observed only in the nasal sector of the RE. OCTA imaging of the optic disc demonstrated normal structure and vascularization.

After pupil dilation, full-field ERG tests were performed following 20 min of dark adaptation for the dark-adapted ERG and 10 min of light adaptation for the light-adapted ERG. Marked reductions in both a-wave and b-wave amplitudes were observed across scotopic, mesopic and photopic responses (Supplementary Figure S1). She underwent clinical genetic testing but parallel sequencing using a comprehensive IRD gene panel (Supplementary Table S1) failed to identify any putative pathogenic or likely pathogenic variants.

### 2.2. Genomic Analyses

The proband was enrolled in a research program dedicated to undiagnosed patients affected with retinal diseases at the Istituto Superiore di Sanità, Rome. Following signed informed consents were secured, peripheral blood samples were collected from the proband, her apparently healthy parents and brother, and purified genomic DNA specimens were used for trio-based WGS. For the proband, mother, and father, the median depth and 20× coverage were 31× and 92.10%, 34× and 94.16%, and 30× and 88.0%, respectively. Reads alignment to the reference genome (hg38) and variant calling and annotation were performed using an in-house implemented pipeline [29]. For variant filtering and prioritization, the analysis initially focused on rare/private single nucleotide variants (SNVs), copy number variants (CNVs), and structural variants (SVs) affecting genes previously involved in IRDs (https://retnet.org (accessed on 14 January 2024)). Subsequently, the analysis was expanded to include all coding genes, revealing compound heterozygosity for two functionally relevant missense variants in the *BAIAP3* gene (Supplementary Figure S2). The first variant, c.556C>G (NM_003933.5, chr16:1340964; rs778671265), predicting the p.Arg186Gly amino acid substitution, was maternally transmitted. The variant had previously been reported in public databases at low frequency in the general population (gnomAD v4.0, MaxAF = 6.950 × 10^−7^, heterozygous occurrence in all cases). The second variant, c.3099C>G (chr16:1347790; rs185896119), resulting in the p.His1033Gln change, was paternally inherited. The variant had been annotated at low frequency in public databases (gnomAD v4.0, MaxAF = 0.000006875 and not reported at the homozygous state). The healthy brother was found heterozygous for the c.3099C>G change. In silico prediction tools provided discordant assessments of the functional consequences of the two missense variants on protein function (Supplementary Table S3).

### 2.3. Functional Studies

**Figure 2:**
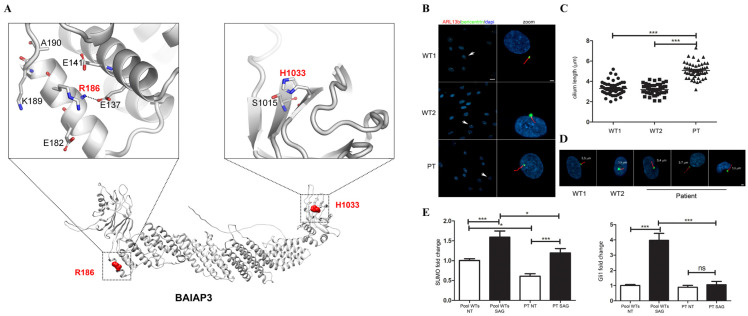
*BAIAP3* variants cause an anomalous primary cilium morphology and induce an aberrant activation of SHH pathway (**A**) Structural model of the wild-type BAI1-associated protein 3 (AlphaFold DB entry: O94812). The protein backbone is shown in gray. Nitrogen and oxygen atoms relative to lateral chains of relevant amino acid residues are in blue and red, respectively. Location of residues Arg186 and His1033, within the *N*-terminal and *C*-terminal C2 domains, are shown. Salt-bridging between Arg186 and Glu137 is highlighted (black dashed line). (**B**) Confocal images showing the altered primary cilium morphology in patient-derived fibroblasts compared to control cells. Primary cilia are labeled with ARL13B (red), while basal bodies and nuclei are labeled with pericentrin (green) and DAPI (blue), respectively. Scale bar is 20 µm left and 2 µm right (zoomed images). (**C**) The scatterplot representing the cilium lengths measured in μm documents the occurrence of statistically significant longer cilia in patient-derived fibroblasts compared to control cells. Cells were analyzed for each line over three independent experiments (30 cells/line each) for a total of 60 cells/line scored. *p* values were calculated by one-way ANOVA with Tukey’s correction for multiple testing. Graph bars show mean ± SEM. (**D**) Representative images of the cilium morphology observed in patient-derived and control cells. Scale bar is 2 µm. (**E**) Graphed data indicating the fold change of target genes of the SHH pathway (*GLI1* and *SMO*) in fibroblasts from the patient carrying biallelic *BAIAP3* variants compared to control cells. Fibroblasts were serum starved for 48 h and treated with Smo agonist (SAG) 100 nM for 24 h. The pool of the controls is set as 1. *GAPDH* mRNA was used as endogenous control. Histograms show mean values ± SEM of two independent experiments, each performed in triplicate. The analysis of the expression was performed calculating the fold change using the 2^−ΔΔCt^ formula and the results were statistically analyzed by PRISM7. *p* values were calculated by one-way ANOVA with Tukey’s correction for multiple testing. Graph bars show mean ± SEM. ns = not significant; *** = *p* < 0.001; * = *p* < 0.1.

The two affected residues, Arg^186^ and His^1033^, are located within the *N*-terminal and *C*-terminal C2 domains of BAIAP3 (*N*-C2; *C*-C2), respectively. The C2 domain is thought to mediate calcium-dependent phospholipid binding and subcellular localization. According to the AlphaFold structural model (entry O94812), Arg^186^ is comprised within the α-helix upstream of the eight-stranded β-sandwich of the *N*-C2 and is spatially close to a neighboring helix outside the domain encompassing two glutamic acid residues, namely Glu^137^ and Glu^141^ (Figure 2A). Of note, Arg^186^ is predicted to establish a salt-bridge interaction with Glu^137^. Hence, this arginine residue likely stabilizes its structural vicinity through H-bonding and electrostatic interactions, and the introduction of the nonpolar glycine residue is predicted to perturb the relative conformation of the interacting helices, locally. Conversely, His^1033^ is not predicted to establish side-chain interactions, and the change to glutamine does not exert visible effects. However, this histidine residue is comprised within the second strand of the *C*-C2 β-sandwich, just upstream of the C2 conserved core, a four-strand Greek-key motif designated as C2 key [30]. Ca^2+^ ions are known to bind in aspartate-rich structural depressions formed by the loops of the C2-key motif. Therefore, being close to the calcium-binding C2 core, the His-to-Gln substitution at codon 1033 is expected to destabilize the domain structure and hinder its calcium-dependent functions. Western blot analysis was performed on patient-derived skin fibroblasts documenting comparable endogenous BAIAP3 protein levels with control cells, ruling out a major impact of both amino acid substitutions on protein stability (Supplementary Figure S3).

**Figure 3:**
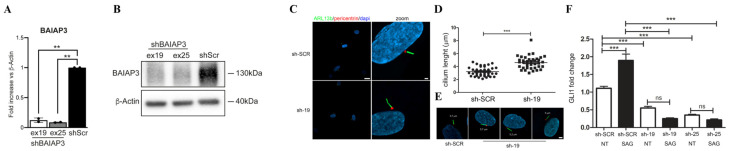
BAIAP3 knockdown in primary fibroblasts is associated with a significant increase in cilia elongation and significant reduction of *GLI1* mRNA expression. Representative western-blot (**A**) and qPCR analysis (**B**) of *BAIAP3* RNAi experiment performed in control (sh-Scr) and *BAIAP3* (ex.19 and ex.25) stably depleted fibroblast cells. β-actin was used as loading control. Scr = scramble; ex = exon. The bars represent mean and SD of two biological replicates; statistical value is reported as ** *p* < 0.01. (**C**) Confocal images showing an altered primary cilium morphology in fibroblasts obtained from a healthy donor in which the *BAIAP3* expression was abolished by interfering compared to the same cell line infected with an empty vector (control cell-sh-SCR). We analyzed by immunofluorescence the fibroblasts in which the *BAIAP3* knockdown was achieved using an interfering RNAs targeting exon 19 (sh-19). In this cell line, we observed a longer primary cilium than control cells. Primary cilia are labeled with ARL13B (green), basal bodies and nuclei are labeled with pericentrin (red) and DAPI (blue), respectively. Scale bar is 20 µm left and 2 µm right (zoomed images). (**D**) The scatter-plot represents the cilium lengths measured in μm. The sh-19 cells show a longer cilium than control. Cells were analyzed for each line over two independent experiments (20 cells/line each) for a total of 40 cells/line scored. Horizontal lines represent the mean of two independent experiments for each subgroup assessed by Mann–Whitney U test (*p* < 0.001). (**E**) In the panel were reported representative images of cilium morphology observed in sh-19 and control cells. Scale bar is 2 µm. (**F**) Data are graphed indicating the fold change of *GLI1*, a target gene of the SHH pathway in sh-19 cells and also in another cell line in which the *BAIAP3* knockdown was achieved using an interfering RNAs targeting exon 25 (sh-25), compared to control cells. Fibroblasts were serum starved for 24 h and treated with SAG 100 nM for additional 24 h. The control (sh-SCR) is set as 1. GAPDH was used as endogenous control. Histograms show mean values ± SEM of two independent experiments, each performed in triplicate. The analysis of the expression was performed calculating the fold change using the 2^−ΔΔCt^ formula and the results were statistically analyzed by PRISM7. *p* values were calculated by one-way ANOVA with Tukey’s correction for multiple testing. Graph bars show mean ± SEM. ns = not significant *** *p* < 0.001.

Based on the well-documented role of the SNARE-mediated trafficking in ciliogenesis, we hypothesized that the two identified variants in *BAIAP3* could be responsible for photoreceptor degeneration and death. To validate this hypothesis, we analyzed the primary cilium biogenesis and morphology in starved primary fibroblasts derived from the affected subject. While the percentage of ciliated cells was comparable in patient-derived and control cells, fibroblasts exhibited an aberrant primary cilium structure. Specifically, we observed a significantly higher number of elongated cilia compared to primary fibroblasts obtained from healthy donors (Figure 2B–D). Ciliary trafficking plays a crucial regulatory role in SHH signal transduction. Based on these considerations, we explored occurrence of altered SHH signaling by assessing the expression of SHH-responsive genes, *GLI1* and *SMO*. Real-time PCR analyses revealed a significant reduction of both *GLI1* and *SMO* mRNA expression level in patient’s fibroblasts compared to control cells, both under basal conditions and following treatment with SAG, a SMO agonist and a SHH pathway activator, indicating aberrant SHH pathway activation (Figure 2E). To further confirm the causal link between loss of BAIAP3 function and the observed endophenotypes, we depleted *BAIAP3* mRNA in primary skin fibroblast obtained from a healthy donor. *BAIAP3* knockdown was achieved using two interfering RNAs targeting exon 19 and 25. Efficient *BAIAP3* silencing in infected fibroblasts was confirmed by qPCR and Western blot analysis (Figure 3A,B). Consistent with previous findings in patient-derived cells, confocal immunofluorescence analysis performed on *BAIAP3*-depleted fibroblasts (sh-19) revealed a significant increased cilia elongation, compared to the control (sh-Scr) (Figure 3C–E), as well as a significant reduction of *GLI1* mRNA expression, both under basal conditions (Figure 3F).

## 3. Discussion

RP embraces a group of IRD primarily characterized by the degeneration of rod and cone photoreceptors. RP is a leading cause of visual disability, and most cases of RP are non-syndromic, typically manifesting during adolescence or adulthood with night blindness, followed by concentric visual field loss, reflecting predominant rod photoreceptor dysfunction. Central vision loss due to cone dysfunction generally occurs later in life. Rod photoreceptor function, as measured by ERG, is markedly reduced or even absent. SD-OCT and FAF macular imaging reveal progressive loss of the outer retinal layers and slow changes of the retinal pigmented epithelium integrity.

Over the past three decades, disease-causing variants in more than 80 genes have been associated with non-syndromic RP [4]. Despite these advances, almost half of RP patients still remain without a molecular diagnosis. Here, we provide evidence that *BAIAP3* is a novel gene contributing to RP when mutated. Biallelic variants in *BAIAP3* were identified in 1 out of 43 IRD individuals who had remained molecularly undiagnosed after comprehensive gene panel analyses for inherited ocular diseases, and were reanalyzed by WGS using a trio-based approach. While our findings suggest that mutations in *BAIAP3* represent a previously unappreciated cause of RP, the relatively small size of the studied cohort does not allow us to provide an accurate estimate of the prevalence of these lesions as cause of RP. The identification of such variants is not entirely unexpected, given that retinal degeneration is a common feature of ciliopathies [31]. Notably, approximately 25% of retinal degenerations are associated with defects in genes involved in the structure and/or function of the photoreceptor cilium [32], highlighting the importance of the ciliary machinery in retinal homeostasis.

BAIAP3 is a paralog of Munc13-4, and its function has not been fully characterized yet. The protein interacts with the SNARE protein complex in a Ca^2+^-dependent manner to mediate endocytic retrograde trafficking and recycling of dense-core vesicles (DCVs) to the trans-Golgi network marker trans-Golgi network 46 (TGN46) [33]. Given the critical role of DCVs in synaptic signaling homeostasis, dysfunction of DCVs is highly likely to result in neuronal abnormalities. Tethering factors and SNARE proteins regulate the final steps of vesicular trafficking by mediating tethering, initial interaction, and fusion of transport vesicles with target membranes, respectively. The Golgi-associated retrograde protein (GARP) complex regulates retrograde transport from endosomes to the TGN. Proteins involved in this regulation, such as the GARP tethering complex, contain a CATCHR domain, which is also present in BAIAP3 [24,25,26,27]. Accordingly, BAIAP3, like GARP, interacts with specific SNARE proteins, including STX6, STX16, VAMP3 and VAMP4 [26]. The mammalian GARP complex interacts with the endosome-TGN SNARE complex (STX16/STX6/VTI1A/VAMP4), and GARP knockdown causes decreased formation and/or stability of this complex [27]. The identified *BAIAP3* variants were not found to affect the levels of the protein in primary skin fibroblasts obtained from the affected individual. However, the collected data, documenting the same altered cilium morphology and impaired SHH signaling in patient-derived cells and *BAIAP3*-depleted control fibroblasts, provide evidence of endophenotypes specifically resulting from the defective function of the protein. It is plausible that functional dysregulation of BAIAP3 may disrupt the endosome-TGN SNARE complex, thereby impairing vesicle trafficking and the delivery of associated proteins required for proper cilium formation and function.

## 4. Material and Methods

### 4.1. Nucleic Acids Extraction

Following the manufacturer’s instructions, genomic DNA (gDNA) was extracted from peripheral blood (PB) samples from the parents and proband using the QIAamp Midi Kit (Qiagen, Hilden, Germany). Total RNA was extracted from PB specimens and purified using a PAXgene Blood RNA kit (PreAnalytiX, QIAGEN, Germantown, MD, USA), as per the manufacturer’s instructions. The RNeasy kit (QIAGEN, Hilden, Germany) was used to extract total RNA from skin fibroblasts, following the manufacturer’s instructions.

### 4.2. Reverse Transcription (RT) Quantitative PCR (q-PCR)

Poly(A) RNA was reverse-transcribed using oligo(dT) primers and SuperScript III Reverse Transcriptase (Thermo Fisher Scientific, Waltham, MA, USA) according to the manufacturer’s instructions. Total RNA was reverse-transcribed using the SensiFAST cDNA Synthesis Kit (Bioline Meridian Bioscience Inc., Memphis, TN, USA). Detection of *GLI1*, *SMO*, *BAIAP3* and *GAPDH* gene expression levels was performed using a qPCR system with SensiMix SYBR Low-ROX Kit (Bioline Meridian Bioscience Inc., Memphis, TN, USA), according to the manufacturer’s instructions. Primer pairs are available upon request. qPCR assayswere performed using an Applied Biosystems 7500 Fast qPCR machine (Thermo Fisher Scientific, Waltham, MA, USA). The fold change of each gene was determined using the 2^−ΔΔCt^ method. Each sample was run in triplicate, in at least two independent experiments.

### 4.3. Whole Genome Sequencing

WGS was carried out using a 2 × 150 bp paired-end read procedure using a NovaSeq 6000 platform (Illumina, San Diego, CA, USA). In each run, a 30-fold median coverage per genome was attained. Bcl2FASTQ was used for base calling and data processing (Illumina, San Diego, CA, USA) [34]. Paired-end read mapping to the GRCh38 reference sequence, variant calling, and joint genotyping were performed using Sentieon v.2023-08 (https://www.sentieon.com (accessed on 10 January 2024)). The Genome Analysis Tool Kit (GATK) v.3.8.0 (Broad Institute, Cambridge, UK) was used to apply hard filtering in accordance with best-practice pipeline guidelines [35,36]. Using the in-house WGS population-matched database (>350 WGS), high quality SNPs and short insertions/deletions (indels) were first filtered by frequency ≤ 5%. Using Sentieon v.2023-08 (https://www.sentieon.com (accessed on 10 January 2024)), distinct in-house-developed procedures were used to annotate and prioritize the remaining low-frequency coding and non-coding variants [37,38]. Variants within the coding regions were annotated and filtered against both in-house (>3100 population-matched exomes) and public (gnomAD 3.1.2, https://gnomad.broadinstitute.org (accessed on 10 January 2024)) variant databases in order to retain rare and private (no frequency or MAF < 0.1%) variants with predicted effecton the coding sequence and within splice site regions. Clinical relevance of variants followed the ACMG 2015 criteria [39], and their predicted functional relevance was examined using the Combined Annotation Dependent Depletion (CADD) v.1.6, M-CAP v.1.3, and InterVar v.2.2.2 algorithms [39,40,41]. Genomiser [42] (phenotype data version 2406) was used to annotate and prioritize variants in non-coding regions. AnnotSV v.3.4.2 [43] was used to annotate and prioritize the structural variations that were detected using paired-end and split-read evidence by DELLY v.1.2.6 [44]. Supplementary Table S2 includes WGS and variant calling metrics.

### 4.4. Structural Analysis

The structural impact of the disease-associated missense changes reported in this study, namely p.Arg186Gly and p.His1033Gln, was assessed using the three-dimensional structure of the wild-type BAI1-associated protein 3, generated by AlphaFold 3 (AlphaFold DB entry: O94812, https://alphafold.ebi.ac.uk/entry/O94812 (accessed on 6 February 2025)) [45]. The structure was visualized and analyzed using the UCSF Chimera software v.1.17.3 (https://www.cgl.ucsf.edu/chimera/ (accessed on 6 February 2025)) [46] and the Mol* web-based toolkit (https://molstar.org/ (accessed on 6 February 2025)) [47]. The side-chain orientations of the residues introduced to simulate the aminoacidic substitutions were obtained from the Dunbrack backbone-dependent rotamer library [48], choosing the best rotamer with minimal to no steric clashes with neighboring residues.

### 4.5. Cell Culture

Functional characterization of BAIAP3 variants was carried out on cultured skin fibroblasts obtained from subcutaneous biopsies of the enrolled patient and controls. Primary fibroblasts were cultured in Dulbecco’s modified Eagle’s medium supplemented with 10% heat-inactivated fetal bovine serum (Gibco) and 1% penicillin-streptomycin, at 37 °C with 5% CO_2_.

### 4.6. Stable Short Hairpin RNAs and Plasmid Transfection

Short hairpin (shRNA) sequences targeting BAIAP3 exon 19 (TRCN0000127610) and exon 25 (TRCN0000416328) and non-Target control shRNA (sh-scr) were obtained from Merck/Sigma-Aldrich (Sigma Aldrich, St. Louis, MI, USA) and sub-cloned into pLKO.1 puro plasmid (#8453 Addgene, Watertown, MA, USA) using AgeI and EcoRI as restriction sites. For lentiviral particles production, constructs were transfected in the presence of pCMV-dR8.2-dvpr and pCMV-VSV-G helper plasmids into HEK293T cells using polyethylenimine (Sigma Aldrich, St. Louis, MI, USA). After 48 h, the supernatant containing lentiviral particles was collected and centrifuged. Primary fibroblast cells were transduced with the supernatant of lentiviral particles in the presence of polybrene (8 μg/mL) for 24 h before replacement with fresh growth medium supplemented with puromycin (1 μg/mL). Cells were analyzed 96 h post transduction.

### 4.7. Western Blot Analysis

BAIAP3 endogenous level was evaluated on fibroblasts lysates, extracted in RIPA buffer (50 mM Tris HCl pH 8, 150 mM NaCl, 1% NP-40, 0.5% sodium deoxycholate, 0.1% SDS.) All samples were resuspended in 4× SDS sample buffer and processed for Western blot (WB) analysis, membranes were probed with a rabbit polyclonal anti-BAIAP3 antibody (#256003,Synaptic Systems, Goettingen, Germany) and an anti-GAPDH antibody (#32233, Santa Cruz Biotechnology, Inc., Dallas, TX, USA) for protein normalization, both diluted 1:1000.

### 4.8. Confocal Analysis

Confocal analysis was performed on a a Zeiss LSM 980 (Carl Zeiss Microscopy GmbH, Jena, Germany) with Airyscan2, using the 63× oil objective and excitation spectral laser lines at 405, 488, 546, 594 and 633 nm.

### 4.9. Primary Cilium Staining

Cells were plated onto cover slips, maintained 24 h in low serum medium to promote emission of cilia and then fixed in 4% paraformaldehyde (PFA). Primary cilium was stained with a rabbit polyclonal anti-ARL13B antibody (#136648, Abcam, Cambridge, UK) followed by goat anti-rabbit Alexa Fluor 594 (red), while basal bodies were stained with mouse monoclonal anti-pericentrin (#28144, Abcam, Cambridge, UK) followed by goat anti-mouse Alex Fluor 488 (green) and nuclei with DAPI (blue). The length of cilia was measured using the Zen Blue 3.3 software (Carl Zeiss Microscopy GmbH, Jena, Germany).

### 4.10. Statistical Analysis

Statistical analysis and post hoc tests were carried out as reported in Figure legends using GraphPad Prism 5.01 (Graphpad Software Inc., La Jolla, CA, USA). In the experiment of confocal microscopy performed after interference of the *BAIAP3* gene, statistical analysis was carried out using the Mann–Whitney U test.

## 5. Conclusions

Collectively, our findings provide evidence that biallelic variants of *BAIAP3* cause photoreceptor degeneration and underlie isolated RP. While *BAIAP3* is highly expressed in various ciliated cells, we observed a clinical phenotype restricted to the retina. We speculate that this might be related to a possible retained function of the protein and a hypomorphic behavior of the two identified missense changes. Retinal homeostasis may be particularly sensitive to defective BAIAP3 function, ultimately resulting in photoreceptor loss. We anticipate the possibility that loss-of-function variants of the gene causing complete inactivation of BAIAP3 might result in a more complex and severe syndromic disorder.

## Data Availability

Raw WGS data are not available due to privacy reasons. All the other data are included in this article and Supplementary Materials. Further inquiries can be directed to the corresponding authors.

## Supplementary Materials

The following supporting information can be downloaded at: https://www.mdpi.com/article/10.3390/ijms26178244/s1.
