# Supplementary material for: Biallelic BAIAP3 Variants Are Associated with Isolated Retinitis Pigmentosa
Source: Int J Mol Sci. 2025 Aug 25;26(17):8244. doi: 10.3390/ijms26178244 (PMC12428663; doi:10.3390/ijms26178244)
Supplement: Supplementary file 1 [file ijms-26-08244-s001.zip › ijms-3807499-supplementary.pdf]

Supplemental Table S1. List of IRD-associated genes originally screened in the affected individual.

Gene	Associated phenotypes	Inheritance
ABCA4	Stargardt disease, Retinitis pigmentosa, Cone rod dystrophy, Retinal dystrophy, early-onset severe, Fundus flavimaculatus	AR
ABHD12	Polyneuropathy, hearing loss, ataxia, retinitis pigmentosa, and cataract	AR
ACO2	Optic atrophy	XL
ADAM9	Macular degeneration/Cone dystrophy	AD
ADGRA3	Retinitis pigmentosa	AR
ADGRV1	Usher syndrome type 2C	AR
AFG3L2	Optic atrophy	AD
AGBL5	Retinitis pigmentosa 75	AR
AGK	Sengers syndrome	AR
AHI1	Joubert syndrome	AR
AHR	Microphthalmia/Anophthalmia	AD
AIPL1	Retinitis pigmentosa, Cone rod dystrophy, Leber congenital amaurosis	AR
ARHGEF18	Retinitis pigmentosa 78	AR
ARL2BP	Retinitis pigmentosa with or without situs inversus	AR
ARL3	Retinitis pigmentosa, Joubert syndrome	AD/AR
ARL6	Bardet-Biedl syndrome, Retinitis pigmentosa	AR
ARSG	Usher syndrome type	AR
ASB10	Primary open-angle glaucoma	AR
ATF6	Achromatopsia	AR
BBS1	Bardet-Biedl syndrome	AR
BBS2	Bardet-Biedl syndrome, Retinitis pigmentosa	AR
BEST1	Vitreoretinochoroidopathy, Microcornea, Rod-cone dystrophy, Posterior staphyloma, Bestrophinopathy, Vitelliform macular dystrophy, Cataract, Retinitis pigmentosa, Macular dystrophy, vitelliform, adult-onset, Retinitis pigmentosa 50, Macular dystrophy, vitelliform 2, Best macular dystrophy, Bestrophinopathy, autosomal recessive	AD/AR
C12ORF65	Optic atrophy	AR
C1QTNF5	Late-onset retinal degeneration	AD
C21ORF2	Retinal dystrophy with or without macular staphyloma (RDMS), Spondylometaphyseal dysplasia, axial (SMDAX)	AR
C2ORF71	Retinitis pigmentosa	AR
C8ORF37	Retinitis pigmentosa, Cone rod dystrophy, Bardet-Biedl syndrome 21	AR
CA4	Retinitis pigmentosa 17	AD
CABP4	Congenital stationary night blindness type 2B	AR
CACNA1F	Aland Island eye disease, Cone rod dystrophy, Night blindness, congenital stationary	XL
CACNA2D4	Cone-rod dystrophy	AR
CDH23	Usher syndrome type 1D	AR
CDHR1	Retinitis pigmentosa, Cone rod dystrophy	AR
CEP290	Bardet-Biedl syndrome, Leber congenital amaurosis, Joubert syndrome, Senior-Loken syndrome, Meckel syndrome	AR
CEP78	Cone-rod dystrophy	AR
CERKL	Retinitis pigmentosa	AR
CHM	Choroideremia	XL
CHM#	Choroideremia	XL

CIB2	Usher syndrome type 1J/Deafblindness	AR
CISD2	Wolfram syndrome type 2	AR
CLN3	Neuronal ceroid lipofuscinosis, type 3	AR
CLRN1	Retinitis pigmentosa, Usher sydnrome, type 3A	AR
CNGA1	Retinitis pigmentosa	AR
CNGA1#	Retinitis pigmentosa	AR
CNGA3	Achromatopsia type	AR
CNGB1	Retinitis pigmentosa	AR
CNGB3	Macular degeneration, juvenile, Achromatopsia	AR
CRB1	Retinitis pigmentosa, Pigmented paravenous chorioretinal atrophy, Leber congenital amaurosis	AR
CRX	Cone rod dystrophy, Leber congenital amaurosis	AD/AR
CTNNA1	Macular dystrophy, patterned 2	AD
CTNNB1	Familial exudative vitreoretinopathy	AD
CWC27	Retinitis pigmentosa with or without skeletal anomalies (RPSKA)	AR
CYP4V2	Retinitis pigmentosa, Bietti crystalline corneoretinal dystrophy	AR
DHDDS	Retinitis pigmentosa, Developmental delay and seizures with or without movement abnormalities (DEDSM)	AD/AR
DHX38	Retinitis pigmentosa	AR
DRAM2	Cone-rod dystrophy	AR
DYNC2H1	Short -rib thoracic dysplasia with or without polydactyly type 1, Short -rib thoracic dysplasia with or without polydactyly type 3, Jeune asphyxiating thoracic dystrophy, SRPS type 2 (Majewski)	AR/Digenic
ELOVL4	Stargardt macular dystrophy	AD
EMC1	Retinal dystrophy with crystalline deposits	AR
ESPN	Deafblindness	AR
EYS	Retinitis pigmentosa	AR
FAM161A	Retinitis pigmentosa	AR
FLVCR1	Ataxia, posterior column, with retinitis pigmentosa	AR
FSCN2	Macular dystrophy	AD
FZD4	Familial exudative vitreoretinopathy 1	AD
GNAT1	Congenital stationary night blindness 1G	AR
GNAT2	Achromatopsia type	AR
GPR179	Congenital stationary night blindness 1E	AR
GRK1	Congenital stationary night blindness 2A	AR
GRM6	Congenital stationary night blindness 1B	AR
GUCA1A	Cone dystrophy	AD
GUCA1B	Cone dystrophy	AD
GUCY2D	Cone rod dystrophy, Leber congenital amaurosis	AD/AR
HARS1	Usher syndrome type 3B	AR
HGSNAT	Mucopolysaccharidosis (Sanfilippo syndrome), Retinitis pigmentosa	AR
HK1	Retinitis pigmentosa	AR
IDH3A	Leber congenital amaurosis	AR
IDH3B	Retinitis pigmentosa	AR
IFT140	Short -rib thoracic dysplasia with or without polydactyly, Asphyxiating thoracic dysplasia (ATD; Jeune)	AR
IFT172	Bardet-Biedl syndrome	AR
IMPDH1	Retinitis pigmentosa, Leber congenital amaurosis	AD

IMPG2	Retinitis pigmentosa, Vitelliform macular dystrophy	AD/AR
INPP5E	Joubert syndrome, Mental retardation, truncal obesity, retinal dystrophy, and micropenis (MORM syndrome)	AR
IQCB1	Leber congenital amaurosis	AR
KCNJ13	Leber congenital amaurosis 16/Snowflake vitreoretinal degeneration	AR
KIAA1549	Retinitis pigmentosa	AR
KIZ	Retinitis pigmentosa 69	AR
KLHL7	Retinitis pigmentosa, Retinitis pigmentosa 42, Cold-induced sweating syndrome 3	AD/AR
LCA5	Leber congenital amaurosis	AR
LRAT	Retinitis pigmentosa, juvenile, Leber congenital amaurosis, Retinitis punctata albescens, Retinal-dystrophy, early-onset severe	AR
LRIT3	Congenital stationary night blindness 1F	AR
LRP5	Familial exudative vitreoretinopathy	AD
MAK	Retinitis pigmentosa	AR
MERTK	Retinitis pigmentosa	AR
MFN2	Hereditary optic neuropathy	AD
MFRP	Microphthalmia, isolated 5, Nanophthalmos 2, Retinitis pigmentosa, autosomal recessive	AR
MVK	Mevalonic aciduria, Hyper-IgD syndrome, Porokeratosis 3, multiple types	AD/AR
MYO7A	Usher syndrome type 1B	AR
NBAS	Optic atrophy	AR
NDP	Norrie disease/Familial exudative vitreoretinopathy	XL
NEK2	Retinitis pigmentosa	AR
NEK2#	Retinitis pigmentosa 67	AR
NEUROD1	Macular dystrophy	AD
NMNAT1	Leber congenital amaurosis	AR
NMNAT1#	Leber congenital amaurosis	AR
NR2E3	Retinitis pigmentosa, Enhanced S-cone syndrome	AD/AR
NR2F1	Congenital ocular anomalies	AD
NRL	Retinitis pigmentosa, Clumped pigmentary retinal degeneration	AD/AR
NYX	Congenital stationary night blindness 1A	XL
OAT	Gyrate atrophy of choroid and retina	AR
OFD1	Simpson-Golabi-Behmel syndrome, Retinitis pigmentosa, Orofaciodigital syndrome, Joubert syndrome	XL
OPA1	Optic atrophy	AD
OPA3	Optic atrophy	AR/XL
OPN1LW	Color blindness (deuteranomaly)	XL
PCARE	Cone-rod dystrophy	AR
PCDH15	Usher syndrome type 1F	AR
PDE6A	Retinitis pigmentosa	AR
PDE6B	Retinitis pigmentosa, Night blindness, congenital stationary	AD/AR
PDE6G	Retinitis pigmentosa	AR
PDZD7	Usher syndrome (modifier)	AR
PEX1	Heimler syndrome, Peroxisome biogenesis factor disorder 1A, Peroxisome biogenesis factor disorder 1B	AR
PEX2	Zellweger syndrome, Peroxisome biogenesis disorder	AR
PEX7	Refsum disease, Rhizomelic CDP type 1	AR
PHYH	Refsum disease	AR
PITPNM3	Cone-rod dystrophy 5	AD

PLA2G5	Fleck retina, familial benign	AR
POC1B	Cone-rod dystrophy	AR
POMGNT1	Muscular dystrophy-dystroglycanopathy	AR
PRCD	Retinitis pigmentosa	AR
PROM1	Stargardt disease, Retinitis pigmentosa, Cone rod dystrophy, Macular dystrophy, retinal,	AD/AR
PRPF3	Retinitis pigmentosa	AD
PRPF31	Retinitis pigmentosa	AD
PRPF4	Retinitis pigmentosa 70	AD
PRPF6	Retinitis pigmentosa 60	AD
PRPF8	Retinitis pigmentosa	AD
PRPH2	Choriodal dystrophy, central areolar, Macular dystrophy, vitelliform, Retinitis pigmentosa, Retinitis punctata albescens, Macula dystrophy, patterned	AD/AR
RAB28	Cone-rod dystrophy	AR
RAX2	Inherited retinal disease	AR/AD
RBP3	Retinitis pigmentosa	AR
RBP4	Retinal dystrophy, iris coloboma, and comedogenic acne syndrome, Microphthalmia, isolated, with coloboma 10	AD/AR
RCBTB1	Retinal dystrophy with or without extraocular anomalies (RDEOA), Familial exudative vitreoretinopathy	AR
RD3	Leber congenital amaurosis	AR
RDH12	Retinitis pigmentosa, Leber congenital amaurosis	AD/AR
RDH5	Fundus albipunctatus	AR
REEP6	Retinitis pigmentosa 77	AR
RGR	Retinitis pigmentosa	AD/AR
RHO	Retinitis pigmentosa, Night blindness, congenital stationary, Retinitis punctata albescens	AD/AR
RIMS1	Cone-rod dystrophy 7	AD
RLBP1	Newfoundland rod-cone dystrophy, Fundus albipunctatus, Bothnia retinal dystrophy, Retinitis punctata albescens	AR
ROM1	Retinitis pigmentosa 7, digenic	AD/AR
RP1	Retinitis pigmentosa	AD/AR
RP1L1	Occult macular dystrophy, Retinitis pigmentosa	AD/AR
RP2	Retinitis pigmentosa	XL
RP9	Retinitis pigmentosa	AD
RPE65	Retinitis pigmentosa, Leber congenital amaurosis	AD/AR
RPGR	Retinitis pigmentosa, Cone-rod dystrophy, X-linked, 1, Macular degeneration, X-linked atrophic, Retinitis pigmentosa 3	XL
RPGRIP1	Cone rod dystrophy, Leber congenital amaurosis	AR
RS1	Retinoschisis	XL
RTN4IP1	Optic neuropathy	AR
SAG	Retinitis pigmentosa, Oguchi disease	AD/AR
SAMD11	Retinitis pigmentosa	AR
SCAPER	Retinal dystrophy, Retinitis pigmentosa	AR
SCLT1#	Senior-Loken syndrome, Retinal dystrophy	AR
SEMA4A	Retinitis pigmentosa, Cone rod dystrophy	AR
SLC24A1	Congenital stationary night blindness 1D	AR
SLC25A46	Optic neuropathy	AR
SLC7A14	Retinitis pigmentosa 68	AR
SNRNP200	Retinitis pigmentosa	AD/AR

SPATA7	Leber congenital amaurosis, Retitinitis pigmentosa	AR
SPG7	Spastic optic atrophy	AR
SPP2	Retinitis pigmentosa	AD
TIMM8A	Mohr-Tranebjaerg syndrome	XL
TIMP3	Sorsby fundus dystrophy	AD
TMEM126A	Optic neuropathy	AR
TOPORS	Retinitis pigmentosa	AD
TRNT1	Retinitis pigmentosa with sideroblastic anemia	AR
TRPM1	Congenital stationary night blindness 1C	AR
TSPAN12	Familial exudative vitreoretinopathy	AD
TTC8	Bardet-Biedl syndrome, Retinitis pigmentosa	AR
TTLL5	Cone-rod dystrophy	AR
TUB	Retinal dystrophy and obesity	AR
TULP1	Retinitis pigmentosa, Leber congenital amaurosis	AR
UNC119	Cone-rod dystrophy	AR
USH1C	Deafness, Usher syndrome, type IC	AR
USH1G	Usher syndrome type 1G	AR
USH2A	Retinitis pigmentosa 39, Usher syndrome, type 2A	AR
VCAN	Vitreoretinal dystrophy	AD
VPS13B	Cohen syndrome	AR
WDR19	Retinitis pigmentosa, Nephronophthisis, Short -rib thoracic dysplasia with or without polydactyly, Senior-Loken syndrome, Cranioectodermal dysplasia (Levin-Sensenbrenner) type 1, Cranioectodermal dysplasia (Levin-Sensenbrenner) type 2, Asphyxiating thoracic dysplasia (ATD; Jeune)	AR
WFS1	Wolfram syndrome	AR
WHRN	Usher syndrome type 2D	AR
ZNF408	Exudative vitreoretinopathy 6, Retinitis pigmentosa 72	AD/AR
ZNF513	Retinitis pigmentosa	AR

Genes associated with RP that were not originally screened in the affected patient are shown in red. WGS data analysis excluded occurrence of functionally relevant variants in all genes.

Supplemental Table S2. WGS and variant calling metrics.

WGS Metrics						
**SAMPLE**	**mean coverage**	**SD coverage**	**median coverage**	**% >1X**	**% >10X**	**% >20X**
father	29.1	10.0	30	98.0%	96.3%	88.0%
mother	33.2	10.6	34	97.5%	96.4%	94.2%
proband	30.2	9.9	31	97.4%	96.2%	92.1%

VCF Calling Metrics								
**SAMPLE**	**het/hom variant ratio**	**total SNPs**	**annotated in dbSNP**	**novel SNPs**	**% dbSNP SNPs**	**total_indels**	**novel indels**	**% dbSNP indels**
father	1.64	3995523	3981367	14156	99.6%	755941	4427	99.4%
mother	1.73	4047704	4033686	14018	99.7%	771229	4138	99.5%
proband	1.68	4012217	3998189	14028	99.7%	770407	4211	99.5%

Supplemental Table S3. Predicted functional relevance of the identified BAIAP3 variants (NM_003933.5).

	***BAIAP3*:c.556C>G**	***BAIAP3*:c.3099C>G**
**Revel**	Benign (moderate) (0.16)	Uncertain (0.41)
**AlphaMissense**	Benign (moderate) (0.104)	Uncertain (0.53)
**MUT Assesor**	Low deleterious probability (1.36)	Benign (-0.37)
**SIFT**	Benign (0.108)	Uncertain (0.005)
**MutationTaster**	Benign (0)	Deleterious (1)
**FATHMM**	Uncertain (-0.7)	Uncertain (-0.49)
**DANN**	Deleterious (0.99)	Deleterious (0.99)
**CADD_phred v1.6**	Uncertain (18.97)	Uncertain (19.61)
**MetaLR**	Benign (low) (0.26)	Benign (low) (0.25)
**PrimateAI**	Benign (Moderate) (0.29)	Uncertain (0.76)
**BayesDel**	Benign (Moderate) (-0.37)	Benign (Supporting) (-0.27)
**GERP**	Uncertain (2.7)	Benign (Supporting) (0.31)
**GenoCanyon**	Deleterious (0.9)	Deleterious (1)
**fitCons**	Deleterious (0.65)	Deleterious (0.72)

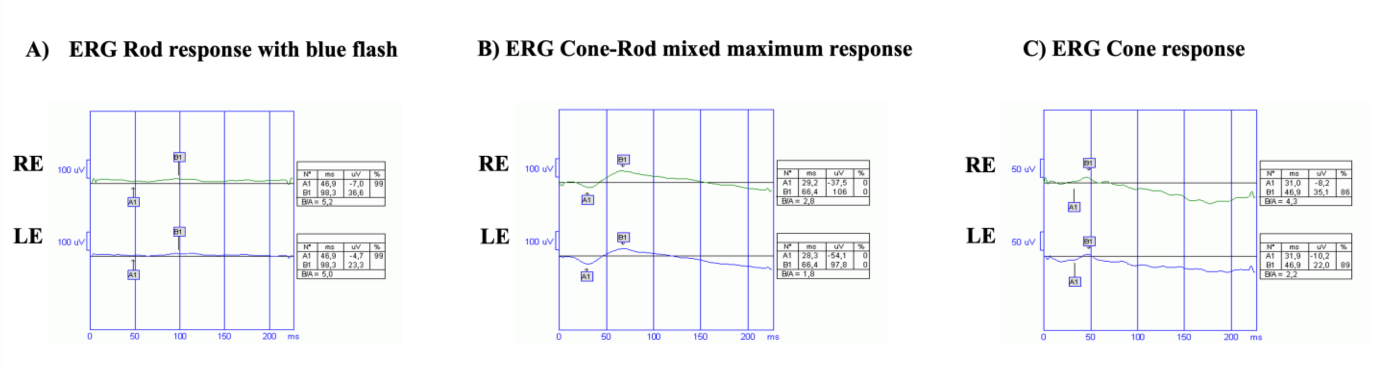

Supplemental Figure S1. Electroretinograms (ERG). ERG recordings were carried out in right eye (RE) and left eye (LE) according to the International Society For Clinical Electrophysiology of Vision (ISCEV) standards. Traces showed marked amplitude reduction of a-wave (A1 peak) and b-wave (B1 peak) of ERG Rod response with blue flash **(A)**, Cone-Rod mixed maximum response **(B)** and Cone response **(C)**. Implicit time was normal for the rods response and increased in the other 2.

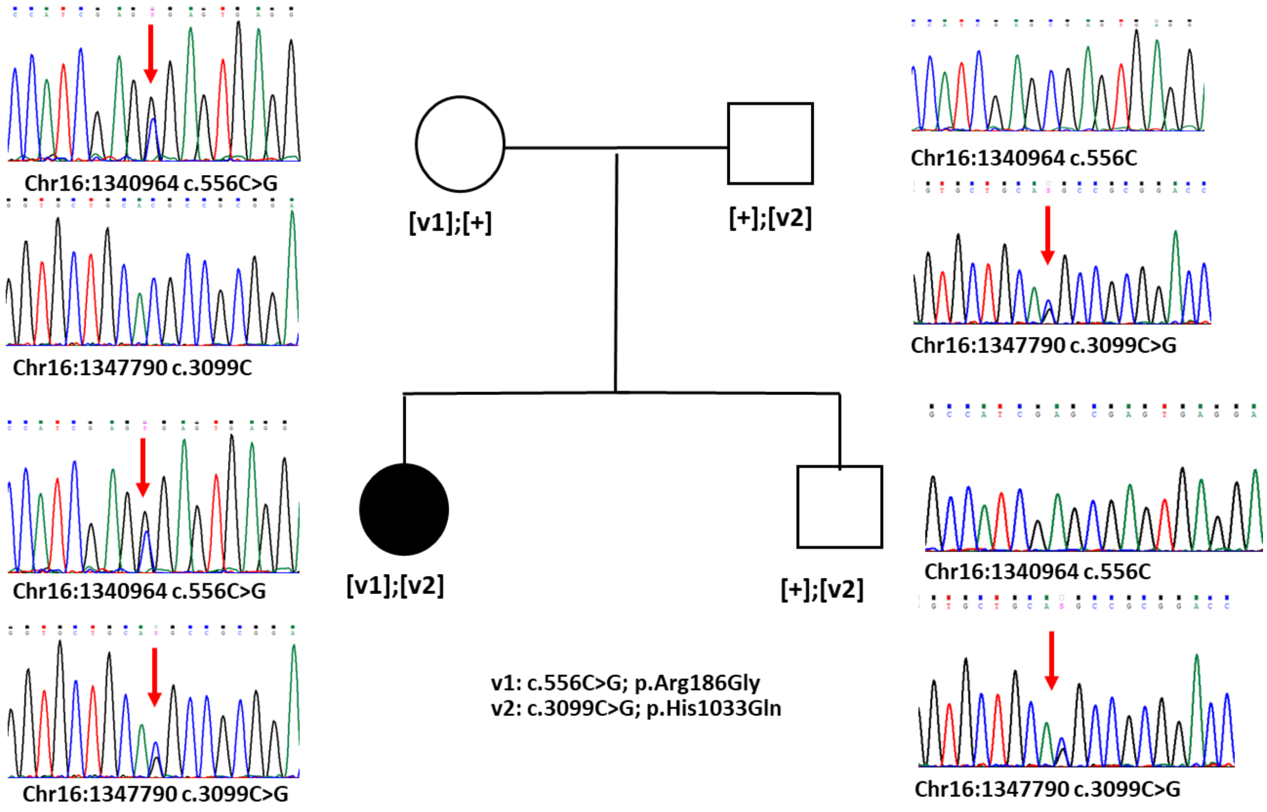

Supplemental Figure S2. Family pedigree chart and segregation analysis. The genotypes of the proband with retinitis pigmentosa, and healthy parents and brother are shown. Chromatograms of the relevant coding stretches encompassing the two missense variants are reported. Heterozygosity for each of the two variants is indicated by a red arrow.

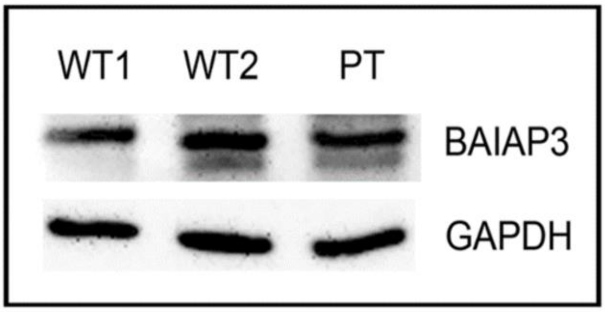

Supplemental Figure S3. Disease-associated *BAIAP3* variants do not affect protein stability. Western blot (WB) analysis showing comparable levels of BAIAP3 in primary fibroblasts from the affected individual (PT) and control cells (WT1 and WT2). Membrane was immunoblotted using a rabbit polyclonal anti-BAIAP3 antibody. The mouse monoclonal GAPDH antibody was used for protein normalization.
